# A dehydration-inducible gene in the truffle *Tuber borchii *identifies a novel group of dehydrins

**DOI:** 10.1186/1471-2164-7-39

**Published:** 2006-03-02

**Authors:** Simona Abba', Stefano Ghignone, Paola Bonfante

**Affiliations:** 1 Dipartimento di Biologia Vegetale dell'Università degli Studi di Torino and IPP-CNR-Sezione di Torino, Viale Mattioli 25, 10125 Torino, Italy

## Abstract

**Background:**

The expressed sequence tag M6G10 was originally isolated from a screening for differentially expressed transcripts during the reproductive stage of the white truffle *Tuber borchii*. mRNA levels for M6G10 increased dramatically during fruiting body maturation compared to the vegetative mycelial stage.

**Results:**

Bioinformatics tools, phylogenetic analysis and expression studies were used to support the hypothesis that this sequence, named TbDHN1, is the first dehydrin (DHN)-like coding gene isolated in fungi. Homologs of this gene, all defined as "coding for hypothetical proteins" in public databases, were exclusively found in ascomycetous fungi and in plants. Although complete (or almost complete) fungal genomes and EST collections of some Basidiomycota and Glomeromycota are already available, DHN-like proteins appear to be represented only in Ascomycota. A new and previously uncharacterized conserved signature pattern was identified and proposed to Uniprot database as the main distinguishing feature of this new group of DHNs. Expression studies provide experimental evidence of a transcript induction of *TbDHN1 *during cellular dehydration.

**Conclusion:**

Expression pattern and sequence similarities to known plant DHNs indicate that TbDHN1 is the first characterized DHN-like protein in fungi. The high similarity of TbDHN1 with homolog coding sequences implies the existence of a novel fungal/plant group of LEA Class II proteins characterized by a previously undescribed signature pattern.

## Background

Hyperosmotic conditions and low temperatures cause cellular dehydration, i.e. removal of water from the cytoplasm into the extracellular space, resulting in the reduction of cytosolic volumes and the alteration of cellular mechanisms. Dehydrins (DHNs) are a group of heat-stable plant proteins believed to play a protective role during cellular dehydration [1,2]. They accumulate during dehydrative stress caused by or associated with low or freezing temperatures, drought, salinity, embryo desiccation and abscissic acid synthesis. Dehydrins are very rich in glycine residues, while cysteine and tryptophane are lacking or under-represented [3]. They are characterized by highly conserved 15-mer lysin rich sequences, called K-segments, which may be present one or several times, one or more Y-segments (DEYGNP) and/or S-segments (serine cluster) [2]. The K-segment can form a putative amphipathic α-helix structure, with the potential for both hydrophilic and hydrophobic interaction [4]. Due to this property, dehydrins potentially have a chaperone-like function in stabilizing partially denatured proteins or membranes, coating them with a cohesive water layer and preventing their coagulation during desiccation [3]. Rinne et al [5] demonstrated that dehydrins could help hydrolytic enzymes maintain their activity even in desiccating environmental conditions, such as freezing. This result confirms the general belief that dehydrins help the cell to survive desiccation, probably creating local pools of water that are required for survival and re-growth.

Dehydrins were initially found in flowering plants, but immunological studies and screenings of cDNA and genome libraries revealed that dehydrins are widely distributed in the plant kingdom [6]. In fact, they were found in the brown algae *Fucus spiralis*, *F. vesciculosus*, and *F. evanescens *[7], in the lichen *Selaginella lepidophylla *[3] as well as in the cyanobacterium *Anabaena sp*. [8]. Dehydrin-homolog sequences are also present in *Escherichia coli *[6] and *Chlamidia trachomatis *[9], and even in *Drosophila melanogaster *[10]. To our knowledge, dehydrins have never been reported in fungi, even if some fungal proteins are classified as late embryogenesis- abundant' (LEA) or LEA-like proteins. Dehydrins belong in fact to this larger protein family. The LEA protein classification proposed by Dure [4] and Bray [11] was recently revised by Wise [12] on the basis of the Kyte and Doolitle hydrophobicity metric, predicted secondary structures, expression patterns and sequence features. Dehydrins are now classified in Class IIa and Class IIb of LEA proteins, corresponding to the previous D11 family or Group 2.

LEA proteins belonging to different classes do not share any evident sequence similarity, even if Garay-Arroyo et al. [13] found that they are characterized by high hydrophilicity and high percentage of glycines, leading to their denomination as "hydrophilins". They are synthesised in the later stages of plant embryogenesis, when seeds are maturing and their water content is decreasing and, in vegetative tissues, in response to water stress [14]. Their precise function is still unknown, but it has been suggested that they are involved in protecting cellular or molecular structures from the damaging effects of water loss by sequestration of ions, replacement of hydrogen bonding function of water or renaturation of unfolded proteins [11,15]. Although primarily found in plants, a number of putative LEA genes have been found in non-plant species, including bacteria [16,17], nematodes [18] and fungi. The first study on a LEA-like protein in fungi was carried out by Mtwisha et al. [14] who suggested that *HSP12 *from *Saccharomyces cerevisiae *should be considered as a LEA-like protein on the basis of its expression pattern and amino acid composition. Also *GRE1 *from *S. cerevisiae *[19] and *CON6 *from *Neurospora crassa *[20] can be ascribed to the family of LEA proteins, because they exhibit a high content of hydrophilic amino acids and their corresponding transcripts accumulate respectively in response to hyperosmosis and desiccation. Moreover, 12 fungal proteins are already classified as 'LEA 4' (named as LEA Class III proteins by Wise [12]) under the Pfam domain family PF02987 on the basis of the presence of at least one IPR004238 (InterPro ID) domain.

In the framework of an expressed sequence tag project aimed at identifying key regulators and master genes controlling the fruiting body formation in the white truffle *Tuber borchii *Vittad. [21], we found that the EST called M6G10 was the most up-regulated gene of the reproductive stage compared to the vegetative stage. Truffles are ectomycorrhizal fungi, producing ascocarps which are highly appreciated and commercialised for their organoleptic properties [22]. Since truffle fruiting bodies cannot yet be obtained under controlled conditions, most studies on truffle primary and secondary metabolism are based on vegetative mycelium cultivated in axenic conditions.

In this study, bioinformatics tools and expression studies were used to support the hypothesis that M6G10 can be considered not only as a LEA protein coding gene, but as the first DHN-like coding gene isolated in fungi. In addition, homologs of this gene, all still defined as "coding for hypothetical proteins" in public databases, were found in other fungal ascomycetous and plant genomes. On the basis of some physiochemical similarities to known plant dehydrins, the identification of a new conserved signature pattern and the expression profile in osmotic and cold stress, we support the classification of these "hypothetical proteins" as dehydrins belonging to Class II LEA proteins.

## Results

### Sequence analyses

**Figure 1:**
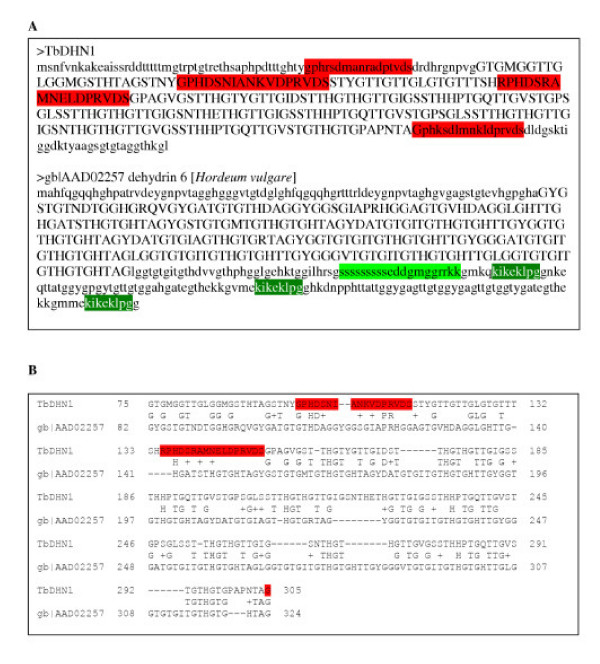
***Panel A*, TbDHN1 deduced amino acid sequence and dehydrin 6 from *Hordeum vulgare***. New CSPs of TbDHN1 (see text for description) [GenBank:DQ308610] are red background coloured; Prosite plant dehydrin signature patterns are green background coloured in dehydrin 6 [GenBank:AAD02257]: S-segment in light green and K-segments in dark green. Sequence fragments aligned by *blastx *are in upper cases (see panel B). ***Panel B*, Basic local alignment between TbDHN1 and dehydrin 6 from *Hordeum vulgare***. The best basic local alignment (E-value = 4e-25) was built by *blastx *between amino acids 75 and 305 of TbDHN1 and amino acids 82 and 324 of dehydrin 6 [GenBank:AAD02257]. New CSPs of TbDHN1 are red background coloured.

The 771-bp-long M6G10 fragment [GenBank:DN601500] was one of the fruiting body-regulated Expressed Sequence Tag (EST) retrieved from a gene expression profiling study conducted in the ascomycetous truffle *T. borchii *[21]. A *blastx *search against NCBI (National Center for Biotechnology Information, NIH, Bethesda) databases [23] using as a query the M6G10 EST revealed the existence of a conserved, previously uncharacterised group of DHN proteins. This result was then confirmed by using the complete *TbDHN1 *mRNA sequence. Significant similarities (E-value < e-10) to other proteins (Table 1), including a plant dehydrin from *Hordeum vulgare *([GenBank: AAD02257]; *blastx *E = 4e-25) (Fig. 1), were found only by masking off repeated or low complexity regions. Dehydrins, like the majority of LEA proteins, are low complexity proteins; in fact, applications masking low complexity regions, like SEG [24], masked between 30% and 71% of the amino acids of a LEA protein [12]. Since the main effect of masking low complexity regions is to reduce the number of amino acids available for alignment, *blast *searches were all performed with the filter for low complexity regions switched off.

A *blastp *search on *Stagonospora nodorum *protein database characterized the predicted protein SNU00161 (The Broad Institute Accession Number) as another possible fungal homolog of TbDHN1. A *tblastx *search was performed querying all those genomic projects in which a proteomic annotation was not yet available. Other possible fungal homologs were thus identified in *Botrytis cinerea *[The Broad Institute Accession Number:00133947_B.cinerea_19866915837883], *Fusarium verticillioides *[The Broad Institute Accession Number:0031597_F.verticillioides_19866917223803], *Chetomium globosum *[GenBank:AAFU01000048], *Coccidioides immitis *[GenBank:AAEC01000142] and *C. posadisii *[TIGR Accession Number:gnl|TIGR_222929|contig:3250:c_posadasii]. A search in the EST databases showed that most of these fungal proteins are present as expressed sequences. Additionally, other DHN-like members were found within cDNA sequences of the fungi *Verticillium dahliae *[GenBank:BQ110173] and *Trichoderma reseii *[GenBank:CF872473], and the plants *Tortula ruralis *[GenBank:CN201388], *Picea engelmannii x Picea sitchensis *[GenBank:DR464575], *Malus x domestica *[GenBank:CV091950], *Oryza sativa *[GenBank:CA765427] and *Saccharum officinarum *[GenBank:CA105075]. For each different species we reported only one entry, because sequences from unfinished/unannotated genome projects and from EST databases are always redundant. Although *blast *searches were performed also on sequences from Basidiomycota and Glomeromycota, all the retrieved fungal sequences belong exclusively to ascomycetous fungi.

Only DHN-like proteins from *T. borchii*, *Gibberella zeae*, *Neurospora crassa*, *Magnaporthe grisea*, *Aspergillus nidulans*, *A. fumigatus*, *S. nodorum *and *Candida albicans *were further analysed. All the other potential fungal and plant DHN proteins were excluded because of poor sequence quality (especially for ESTs), i.e. high percentage of Ns or too short fragments, and because all *tblastx *searches were performed on an unfinished genome project.

Besides sequence similarities, additional evidences supported the relatedness between TbDHN1, the twelve fungal uncharacterized proteins and the big family of LEA proteins (Table 2): a) the amino acid composition (Pepinfo analysis), e.g. richness in Gly and polar amino acids such as Thr and Ser and lack of both Cys and Trp; b) the high percentages of low complexity regions (SEG analysis); c) more than 50% of the polypeptide is predicted to be structured as a random coil (Predictprotein analysis); d) the high hydrophilicity (ProtScale analysis), e.g. maximum hydrophobicity for all sequences is 0.7 in GenBank:EAL87020 from *A. fumigatus*. GenBank:EAA54716 from *M. grisea *is the sole sequence in which cysteine and tryptophan are present, although in a very low percentage. Low complexity regions represented a high percentage of each protein, with 7 sequences showing more than 30% of their amino acids masked by SEG. The exceptions are Swiss-Prot:Q7S6B0 from *N. crassa *(0%) and GenBank:EAL87020 from *A. fumigatus *(5%) which also shared the lowest sequence similarity with TbDHN1.

On the basis of these physiochemical characteristics, TbDHN1 and its fungal homologs can be assigned to the large family of LEA.

### Classification according to Wise's rules

The next step in the characterization of TbDHN1 and its homologs was their classification within the big family of LEA proteins. TbDHN1 and the other twelve fungal proteins were classified according to a set of rules defined by Wise [12]. Each LEA Class is defined by a range of percentages for all the physicochemical features which usually characterize LEA proteins: hydrophilicity, predicted secondary structure and amino acid composition. According to this rule set, *G. zeae*, *A. nidulans*, *A. fumigatus *and *C. albicans *proteins were placed into Class II; while the aromatic amino acid percentage of *N. crassa *and *M. grisea *proteins and the values of minimum hydrophobicity for *T. borchii *and *S. nodorum *proteins were out of the established ranges for this Class. It is important to point out that, especially for *N. crassa*, *M. grisea *and *T. borchii *proteins, values of aromatic amino acid percentage and minimum hydrophobicity are very close to the limit of Class II. The six fungal proteins not assigned to Class II clustered with Class IV, but Wise concluded that members of Class IV should be more appropriately housed in Class II and Class III. According to Wise's analysis of LEA amino acid composition, glycine is highly represented in Class II, while in Class III glycine is found only marginally more than expected by chance. Moreover, Class III LEA proteins have high helix content. On the basis of a high percentage of glycines in the six proteins and/or a lower percentage of helix content than the expected one for Class III, also proteins from *N. crassa*, *M. grisea*, *T. borchii *and *S. nodorum *can be assigned to Class II, the group of plant dehydrins.

Furthermore, *blastp *results obtained with TbDHN1 as query against the nr protein database shared no common hits with the ones obtained using as queries the fungal proteins already classified in Class III (Pfam PF02987).

### A new common signature pattern

**Figure 2:**
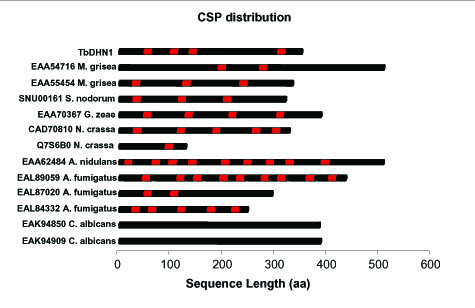
**Distribution of the conserved signature pattern (CSP) within TbDHN1 and its homolog sequences**. CSP is indicated with red boxes.

Members of plant DHN family are characterized by the presence of two PROSITE signature patterns: the S-segment S(5)-[DE]-x-[DE]-G-x(1,2)-G-x(0,1)-[KR](4) (with the exception of pea dehydrins, *Arabidopsis thaliana *COR47 and XERO2 and wheat cold-shock proteins) and the K-segment [KR]-[LIM]-K-[DE]-K-[LIM]-P-G. These common signature patterns are not present in the fungal sequences. However, another new repeated conserved signature pattern (CSP) was identified in sequences from filamentous fungi: [GRK]-[PV]-H-x-[ST]-x-x-x-N-[nonpolar amino acid]-[nonpolar amino acid]-D-P-[RTP]-V-D-[SN]. This deduced signature pattern identified at the C-terminal represents the first block of amino acids aligned by T-Coffee [25] and it is repeated from one to nine times within each sequence with slight variations (Fig. 2). The different number of repetitions of the CSP poses a challenging problem to the alignment of these regions, but T-Coffee aligned all the first and the last repetitions together, because further conserved residues flank these two repetitions. Not only TbDHN1 and the analysed fungal proteins, but all the previously cited fungal and plant sequences retrieved from *blast *searches show this CSP. It has to be underlined that sequences from *C. albicans *do not show this CSP but, in any case, they do share common physicochemical features with the other fungal sequences (Table 2).

As in plant DHNs, in TbDHN1, GenBank:EAA55454 from *M. grisea*, GenBank:EAA70367 from *G. zeae*, SNU00161 from *S. nodorum*, GenBank:CAD70810 from *N. crassa *and both proteins from *C. albicans*, there are repetitions of amino acid strings which are identical within the same sequence but different from one sequence to the other (data not shown).

### Phylogeny, structure and localization prediction

**Figure 3:**
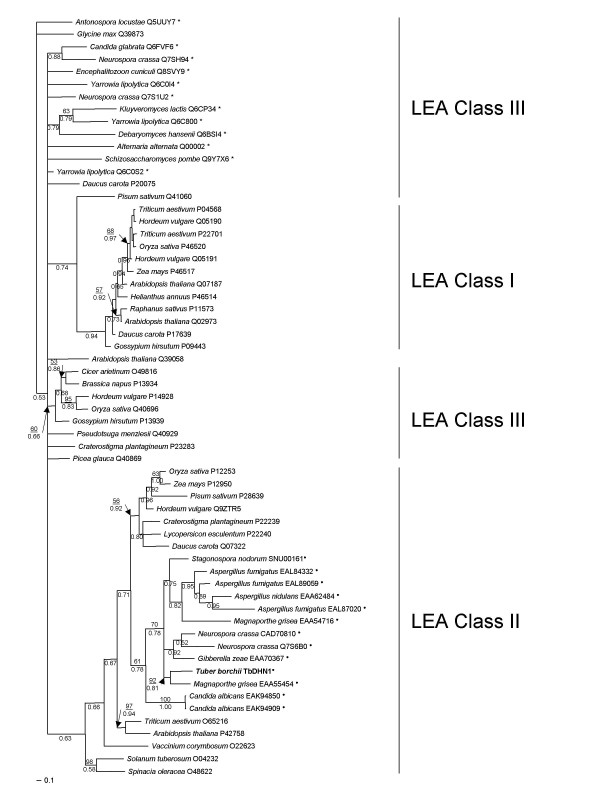
**Bayesian posterior probability tree**. Neighbor-Joining bootstrap values, grater than 50%, are shown above branches where available; Bayesian posterior probabilities are shown below branches. A full circle denotes the newly classified sequences, while the other LEA proteins were already classified by Wise [12]. Fungal LEA Class III proteins are marked with an asterisk. Newly generated TbDHN1 sequence is in bold. Bar = posterior probability.

The sequence alignment among TbDHN1 and its fungal homologs, selected plant LEA Class I, Class II, Class III and fungal Class III proteins (Table 3) generated by T-coffee was used to infer the phylogenetic relationship. NJ (data not shown) and Bayesian posterior probability analyses (Fig. 3) showed a congruent tree topology. Bayesian analyses yielded a phylogenetic tree in which clades corresponding to LEA Class I, Class II, Class III proteins are recognizable, although the LEA Class III sequence group is not well defined. A first clade, supported by a posterior probability value of 0.74, comprises all LEA Class I proteins and a Class III protein from *P. sativum*. A second clade, supported by a posterior probability value of 0.63, includes all LEA 2 proteins from plant, TbDHN1 and its fungal homologs. Fungal LEA Class II proteins clustered together with a posterior probability value of 0.78 and 61% NJ bootstrap value. Despite the lack of CSP, also *C. albicans *proteins are included in the same cluster. Notably, Swiss-Prot:Q9ZTR5_HORVU, the best hit of known function provided by *blastx *searches, falls in this clade. Plant and fungal Class III proteins clustered together but did not form a well separated clade supported by posterior probability and MP bootstrap values higher than 50%.

A multiple alignment among TbDHN1 and its fungal homologs were generated with T-coffee and used to build a Hidden Markov Model profile. This was used to query Swiss-Prot and Pfam databases, but no hit with a significant score was retrieved: the first hit was an Ice nucleation protein (Swiss-Prot:P09815), E-value = 0.076, from *Pseudomonas fluorescens*.

WoLF PSORTII predicted for TbDHN1 and the other twelve fungal proteins a probable nuclear/cytoplasmic localization, but no nuclear localization signals were found by PredictNLS program.

### The case study of TbDHN1: gene structure

**Figure 4:**
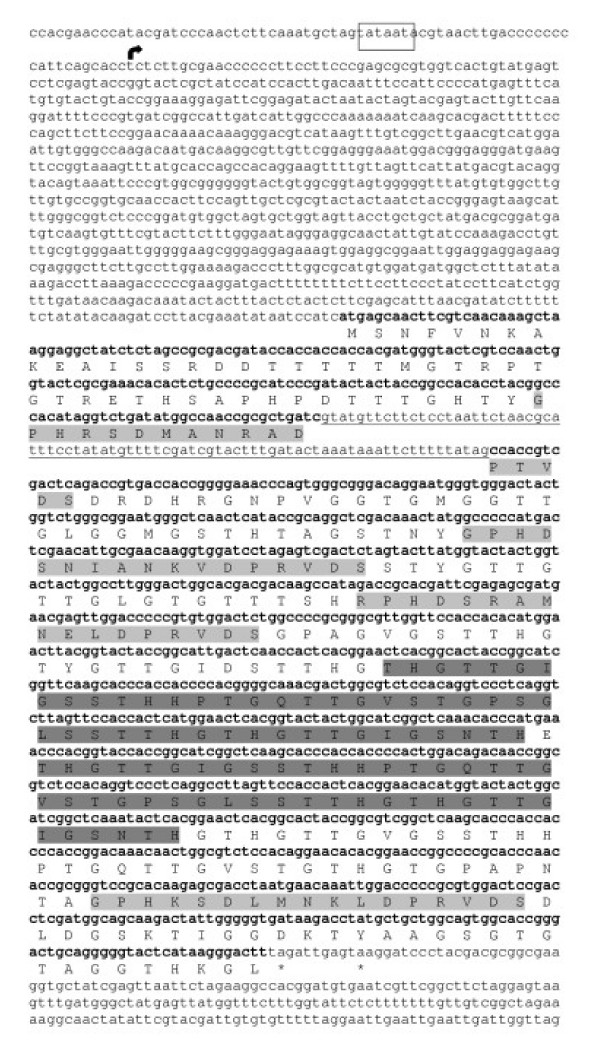
**Nucleotide and amino acid sequences of TbDHN1**. Genomic and mRNA were sequenced by genomic and cDNA library screening. The coding sequence is shown in bold lower case; the 79-bp-long intron is underlined. The putative TATA box is indicated by an open box.  indicates the transcription initiator. Stop codons are marked with an asterisk. AA amino acids that form the CSP; AA amino acids that form the repeated block of 46 amino acids.

The entire sequence of *TbDHN1 *gene was obtained from a *T. borchii *cDNA and genomic library screening. *TbDHN1 *mRNA was a 2300-bp-long open reading frame coding for a predicted 351 amino acid long polypeptide with a predicted molecular mass of 34,8 kDa. The genomic sequence revealed the presence of a 79-bp-long intron after 178 nucleotides from the translation initiator ATG and a putative TATA-box located at position -35 nt from the transcription start site (Fig. 4). Besides the presence of 4 repetitions of the CSP, another peculiar feature has to be pointed out: a block of 46 amino acids is repeated within the sequence, separated only by a glutamic acid residue. The corresponding nucleotide sequences are almost identical with slight variations only in the third nucleotide of some codons (Fig. 4).

**Figure 5:**
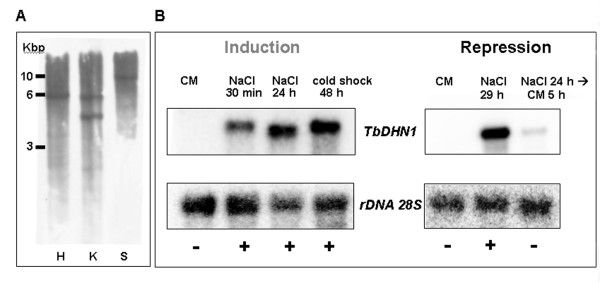
***Panel A*, Southern Blot analysis of *TbDHN1***. *T. borchii *genomic DNA digested with *HindIII *(H, *Lane 1*), *KpnI *(K, *Lane 2*) and *SmaI *(S, *Lane 3*) was probed with an ECL-labelled *TbDHN1 *cDNA probe. ***Panel B*, Gel-blot analysis of the TbDHN1 transcript under salinity and low temperature stress. **Induction (left): mRNA extracted from mycelia grown for 30 days on control medium (CM; *Lane 1*); treated for 30 min in NaCl 0.4 M (NaCl 30 min; *Lane 2*); treated for 24 h in NaCl 0.4 M (NaCl 24 h; *Lane 3*); treated for 48 h at 4°C (cold shock 48 h; *Lane 4*). Repression (right): mRNA extracted from mycelia grown for 30 days on CM (CM; *Lane 1*), treated for 29 h in NaCl 0.4 M (NaCl 29 h; *Lane 2*); treated for 24 h in NaCl 0.4 M and then transferred on CM for 5 h (NaCl 24 h→ CM 5h; *Lane 3*). Signals obtained by ^32^P -labeled *TbDHN1 *probe (top) were normalized using the rRNA 28 S probe as internal standard (bottom).

The genomic region amplified by DHNf/DHNr primers contains one *KpnI *restriction site but no site for *HindIII *and *SmaI*. As revealed by DNA gel blot data reported in Figure 5A, a single-band pattern was produced by hybridization of the *TbDHN1 *probe to genomic DNA digested with *HindIII *and *SmaI *and a double-band was produced by *KpnI*, thus indicating that *TbDHN1 *is encoded by a single copy gene in *T. borchii *genome.

### Stress-regulated expression of TbDHN1

Previous experiments showed that *TbDHN1 *transcript was strongly upregulated in *T. borchii *fruiting bodies compared to vegetative mycelium grown on control medium [21], but, since fruit bodies can be collected only in field, we chose to further investigate *TbDHN1 *expression using vegetative mycelium grown in axenic conditions.

RNA gel-blot experiments were conducted to determine whether *TbDHN1 *transcription levels increase in the same conditions affecting plant DHNs transcription, e.g. low temperature stress and salinity stress. The RNA gel-blot data reported in Figure 5B showed that the *TbDHN1 *messenger increased immediately following transfer on NaCl medium for 30 min and such up-regulation is still evident in a NaCl treatment prolonged to 24 h. The same strong up-regulation response is also evident after a 48 h-long cold treatment. *TbDHN1 *messenger rapidly returned to basal levels when mycelia cultivated for 24 h on NaCl medium were shifted for 5 h on control medium, while in mycelia continually cultivated for 29 h on NaCl medium the up-regulation was still evident.

## Discussion

Almost 40 genomes of fungi have been completely sequenced or are currently in the pipeline according to the NCBI website. In any newly sequenced eukaryotic genome, more than 30–40% of the genes usually do not have an assigned function [26]. Remarkably, a relatively small fraction of the uncharacterized genes is species- or genus-specific; the majority of such "hypothetical" genes have a wider phyletic distribution and therefore are usually referred to as "conserved hypothetical" genes [27,28]. Although it appears that the central pathways of information processing and metabolism are already known, important signalling and stress response mechanisms remain to be studied [29].

In our previous work on key regulators and master genes controlling the morphogenetic events of truffle fruiting body formation, 16 out of the 55 transcripts preferentially expressed in fruit bodies showed significant homology to other fungal hypothetical proteins of unknown function [21]. The availability of experimental data on the upregulation of *TbDHN1 *(M6G10 EST) in *T. borchii *reproductive stage, the presence of TbDHN1 homologs in other ascomycetous fungi and the absence in other fungal lineages (especially in Basidiomycota and Glomeromycota) made this gene a priority target for investigations on ascomycetous fruiting body formation. Conserved genes having a patchy phyletic distribution could be functional determinants of particular phenotypes and let us hypothesize that this DHN-like gene may have been acquired by some organisms after the separation of the main fungal evolutionary lineages which was estimated at 1400 Myr [30]. On the other hand, TbDHN1 and its homologs are supposed to be a part of the larger family of LEA proteins, so we can assess that this protein family has a nearly ubiquitous phyletic distribution: they were already found in plants, algae, invertebrates, bacteria and in ascomycetous fungi. Significant positive correlation between the phyletic spread of a gene and the likelihood that it is essential for cell growth has been demonstrated [31]. Since LEA proteins belonging to different classes have no evident sequence similarity but only common structural and physicochemical characteristics, the hypothesis of an independent evolution in different organisms under dehydration conditions is more likely than the hypothesis that they share a common ancestor [13]. Defence against water deficit caused by low temperature, salinity stress or drought is, in fact, a crucial point in preventing cell damage, especially for those organisms, like plants and microrganisms, that are unable to escape from critical environmental conditions.

Another point of interest raised by TbDHN1 and its homologs bears upon the presence of a repeated conserved signature pattern (CSP) which groups together all the sequences found in filamentous fungi and plants. We supposed that the two proteins from *C. albicans *do not show this CSP because they are from a lievitoid Ascomycete, but we do not exclude that they could share a not-yet identified CSP with other *Saccharomycetales*.

The different number of repetitions of the common signature pattern in various organisms could be explained as a possible consequence of multiple events of internal duplication. Repetitions of the CSP within the same sequence are not exactly identical; therefore, after an initial event of internal duplication, a differentiation of the CSP may have occurred. The fungal CSP, as well as other repeated modules of proteins [32], could be a structural/functional domain.

The presence of the newly identified CSP in the ESTs CN2013881 from *T. ruralis *and CA765427 from *O. sativa *is very intriguing. The first sequence was in fact isolated during a study on the rehydration transcriptome of *T. ruralis *[33], an important plant model for studying plant stress responses and vegetative desiccation tolerance. The second sequence was isolated from an *O. sativa *cDNA library constructed from drought stress panicle. Therefore, the presence of the new CSP in these transcripts suggests that it is associated with transcripts involved in responses to cellular dehydration.

### Why consider TbDHN1 and its homologs as DHN-like proteins?

*In silico *and experimental analyses suggest that TbDHN1 and its homologs should be classified as dehydrins. There is much evidence to support this claim. Firstly, all the protein sequences shared the typical physiochemical properties of the large family of LEA proteins. More precisely, they fitted the set of rules defined by Wise to define Class II, the group of all plant dehydrins, although Wise trained his application with plant proteins exclusively. Class II of plant LEA proteins is also characterized by the presence of two conserved signature patterns. TbDHN1 and its homologs do not have any of the already identified conserved signature patterns, but those proteins from filamentous fungi and plants show a new, previously uncharacterized CSP. This pattern is variously repeated in the fungal sequences: from the single repetition in *N. crassa *Q7S6B0 to the nine repetitions in *A. nidulans *GenBank:EAA62484 and *A. fumigatus *GenBank:EAL89059. Except for the border-line case of *N. crassa *Q7S6B0, the last repetition of CSP at the C-terminal of all the sequences is the most conserved one. The absence of the conserved signature patterns typical of plant dehydrins is not sufficient to exclude a protein from Class II of LEA proteins; in fact Wise includes Swiss-Prot:O22623 from *Vaccinium corymbosum *in Class II, although the sequence lacks both conserved signature patterns.

All this evidence is further supported by the phylogenetic inference: the tree topology shows how TbDHN1 and its fungal homologs cluster together with LEA Class II proteins. *C. albicans *proteins too fall in the same clade, although they lack the fungal CSP.

We can thus determine that TbDHN1 and its homologs are dehydrin-like proteins. EAK94850 (NCBI Protein Database Accession Number) and EAK94909 (NCBI Protein Database Accession Number) from *C. albicans *were already recorded in the protein database at NCBI as "dehydrins", but exclusively on the basis of their homology with DHN6 from *H. vulgare*, the same protein which represents the best hit of the known biological role for TbDHN1. Here, our physiochemical and structural analyses provide further substantial elements to their assignment as dehydrins.

### TbDHN1 gene structure and expression pattern

*In silico *analyses on fungal dehydrin-like proteins were followed by an extensive study on TbDHN1 gene structure and expression pattern in stress vs. control conditions. Southern blot analysis showed that TbDHN1 is a single copy gene but, in the absence of complete *T. borchii *genome sequence data, we cannot exclude the presence of other DHN-like coding genes in *T. borchii*. Since a number of DHN-like genes from the same genome (e.g. *N. crassa*, *M. grisea*, *C. albicans *and *A. fumigatus*) are very different in nucleotide sequence, the *TbDHN1 *probe could be expected to fail to match other DHN-like genes. The presence of multiple DHN-like proteins in a single proteome leads to the hypothesis that they represent a multigenic family; in fact, concerning *A. fumigatus *genome, the three identified DHN-like proteins are localized on three different chromosomes: EAL89059 on chromosome 6, EAL84332 (NCBI Protein Database Accession Number) on chromosome 4 and EAL87020 on chromosome 7.

RNA gel-blot experiments determined that *TbDHN1 *transcription levels increase in the same conditions affecting plant DHNs transcription, e.g. low temperature stress and salinity stress. This strong upregulatory response is fully reversible, since basal levels of the *TbDHN1 *transcript were recovered following additional five-hour incubation on control medium.

The upregulation of M6G10 EST in fruiting body compared to vegetative mycelium [21] is probably strictly linked to the exposure of fruiting bodies to low temperatures during the autumn/winter season. *T. borchii *ascomata develop from November to April and the best harvests generally result from a period of poor growing conditions, such as drought and low temperature, immediately followed by optimal conditions of warmth and moisture [34]. Fruiting bodies analyzed in our previous work were in fact sampled in a natural truffle ground near Alba in Piedmont (Italy) during the December 1999 – March 2000 and December 2000 – March 2001 production seasons, when the average registered temperatures were 5.7°C and 6.3°C, respectively. This may explain why M6G10 EST was the most expressed transcript in fruiting bodies compared to vegetative mycelium grown at 24°C. The high up-regulation of *TbDHN1 *transcript in water-limiting conditions could represent one of the molecular mechanisms to face the threat of desiccation. Many other genes encoding for stress proteins were up-regulated during the reproductive stage [21], including a putative hsp12 [GenBank:CN488002] which shares 31% identity with hsp12 from *S. cerevisiae*. The latter is also supposed to be a LEA-like protein because it is highly expressed when there is an increased osmolality within the cell, such as during spore maturation [14]. It thus seems that in *T. borchii *there is a molecular mechanism involving a set of genes specifically induced in response to cellular dehydration resulting from developmental events like fruiting body formation/maturation or environmental stimuli such as osmotic stress and low temperature. Although a transient transformation of *T. borchii *mycelium has already been obtained [35], stable transformants cannot be exploited to provide a direct functional evidence to support this hypothesis.

## Conclusion

Expression pattern and sequence similarities to known plant DHNs indicate that TbDHN1 is the first characterized DHN-like protein in fungi. The high similarity of TbDHN1 with homolog coding sequences implies the existence of a novel fungal/plant group of LEA Class II proteins. Since most of the DHNs reported so far are from plants, it is not surprising that TbDHN1 displays a conserved repeated signature pattern that distinguishes it from other DHNs. This new signature pattern will be proposed to Prosite as the main feature of this new DHN-like group of proteins and all the new dehydrins could be grouped in the ProDom uncharacterized protein family PD844692, which was so far populated only by the sequence CAD70810 from *N. crassa*.

Another remarkable feature of these DHN-like proteins is their peculiar distribution. In fact, even though complete (or almost complete) genomes and EST collections of some Basidiomycota, Glomeromycota and Viridiplantae are already available, they appear to be represented only in Ascomycota and in a limited number of plant families. In any case it is possible that other homologs of TbDHN1 will be soon identified in other fungi or plants that have only partial genome coverage.

This is not the first time that a new protein has been characterized using bioinformatic and biological investigation of genes/peptides isolated from *T. borchii*. TbSP1 identified a novel family of fungal/bacterial PLA_2_s [36] and, more recently, a *T. borchii *EST was characterized as the first member of the new family of Cyanovirin-N Homology [37]. Taken on the whole, these data suggest that the *T. borchii *genome is a potential goldmine of uncharacterized genes for understanding new aspects of microbial biology.

## Methods

### Sequence isolation

The 771-bp-long M6G10 EST was isolated from an expression library constructed from a mature fruiting body (CF70) of *Tuber borchii *[21]. cDNA array hybridizations showed that this clone was the highest upregulated gene (163.7-fold) between the reproductive and vegetative stage.

Two specific oligonucleotides (DHNf: 5'-GGTACTCGCGAAACACACTCTG-3'); DHNr: 5'-TGGCCTTCTAGAATTAACTCG-3') were used as PCR primers to amplify the corresponding region on M6G10 EST. PCR reaction was performed in a 30 μl volume reaction which consists of 80 μM of each deoxynucleoside triphosphate, 50 pmol of each primer, 1 U of RED*Taq *DNA polymerase (Sigma), and the buffer supplied by the manufacturer of the RED*Taq *DNA polymerase. The PCR parameters were as follows: 94°C for 3 min; 94°C for 45 sec, 60°C for 45 sec, and 72°C for 1 min for 35 cycles; and a final cycle at 72°C for 10 min. The resulting DNA fragment was purified with the QIAquick PCR purification kit (Qiagen) and utilized as a probe for plaque hybridization analysis of two *T. borchii *libraries: a cDNA library of vegetative mycelium grown for 30 days on MMN liquid medium, constructed in the phage vector Uni-Zap XR, and a DNA library constructed in the phage vector EMBL4 (kindly provided by B. Lazzari and A. Viotti, Istituto Biosintesi Vegetali, CNR, Milano). Phage DNA extracted from hybridization-positive plaques of both screenings was sequenced by Genome Express (Meylan – France). The full-length cDNA and the corresponding genomic sequence were thus obtained and deposited to GenBank database [GenBank:DQ308610]. Hereafter we refer to M6G10 complete genomic and mRNA sequence as *TbDHN1*.

### Homology search

Initial homology searches were conducted with M6G10 sequence against the non-redundant (nr) protein sequence and the Swiss-Prot databases at the NCBI by using *blastx *program [38]. To identify homologs within Expressed Sequence Tags collections, *blastn *and *tblastn *searches were run against the EST database at NCBI and the fungal plant pathogen database [39].

Genome and protein sequences (2005-06-31 release) from fungi and plants available at NCBI, the Broad Institute [40] and TIGR [41] were downloaded to a local database and formatted to be used with a local BLAST installation. The complete nucleotide sequence of *TbDHN1 *was used to query public databases using *blastx *and *blastp *programs. All *blast *results were loaded into a MySQL [42] database for further analyses and data mining.

On the basis of sequence similarities, other twelve, previously uncharacterised, fungal TbDHN1 homologs were identified (Table 1). All twelve fungal proteins derived from a conceptual translation of their genome. The following *in silico *analyses were performed on TbDHN1 and these twelve fungal hypothetical proteins.

### Secondary and tertiary structure predictions

Secondary structure percentage composition was obtained by a four-state prediction for each amino acid using PHDsec from ProteinPredict server [43]. The four-state predictions were converted to percentage composition values in order to minimize the effects due to differences in length across the sequences as suggested by Wise [12].

The program T-Coffee [25] was used to build a multiple alignment with *TbDHN1 *and the hits obtained by homology searches (Table 1). The alignment was slightly manually optimized with GeneDoc [44]. Based on the multiple fungal alignment, a profile hidden Markov model (HMM) was built using HMMER [45] in order to search Swiss-Prot and Pfam for a sequence family's consensus.

### Phylogenetic analysis

T-Coffee was used to build a multiple alignment among the TbDHN1 fungal homologs, plant LEA proteins recently classified by Wise [12] in Class I, Class II and Class III and fungal sequences previously reported as LEA proteins by Pfam database (Pfam Database Accession Number PF02987) (Table 3). Among all plant LEAs, twelve proteins were chosen from each LEA class to represent the highest number of different plant species. Only LEA Class I, II and III were considered because they are the only three groups in which conserved consensus sequence motifs were identified. The alignment was slightly manually optimized with GeneDoc.

The Neighbour-joining (NJ) was performed using PAUP* 4.0b10 [46]. Robustness of the internal branches was assayed by bootstrap analysis (1000 runs). Bayesian inference based upon posterior probability distribution of trees was performed with MrBayes [47] with the following settings: seed and swapseed = 1, generations = 1,000,000, runs = 2, chains = 4, sampling every 100^th ^generation, starting trees = random, other settings = default. Phylogenetic trees were rendered with TreeViewX [48].

### Amino acid composition

The EMBOSS application Pepinfo [49] was used to calculate amino acid class (Aliphatic, Aromatic, Non-polar, Polar, Charged, Basic and Acidic) percentage compositions and hydrophobicity values based on the method of Kyte and Doolittle. As regards the determination of hydrophobicity, a 21 aa window was used as suggested by Wise [12]. Three values were considered for each sequence: the minimum, the maximum and the average hydrophobicity; negative hydrophobicity values indicate hydrophilicity.

### Subcellular localization prediction

Subcellular protein localization and cleavage sites predictions were made with the WoLF PSORTII web server [50] selecting "fungi" as organism type; nuclear localization signals were predicted with the PredictNLS program [51].

### Expression and DNA analyses

Expression studies were performed to characterize only *TbDHN1 *gene. *T. borchii *Vittad. mycelia (isolate ATCC 95640) were grown in the dark at 24°C on a synthetic solid medium (control medium, CM) containing CaCl_2_· 2H_2_O (66 mg/L), NaCl (25 mg/L), KH_2_PO_4 _(500 mg/L), (NH_4_)_2_HPO_4 _(250 mg/L), MgSO_4_· 7H_2_O (150 mg/L), FeNa-EDTA (20 mg/L), and thiamine hydrochloride (1 mg/L), plus agar (15 g/L) and glucose (5 g/L). To allow easy medium replacement, myceliar cultures were grown on semipermeable cellophane membranes. To determine whether *TbDHN1 *transcription levels increase in the same conditions affecting plant dehydrin transcription, mycelia were first cultured on control medium at 24°C and then transferred to either a single component-changed media at 24°C in which NaCl was 1000-fold more concentrated than in CM (hereafter designated as NaCl treatment) or to the same control medium at 4°C (hereafter designated as cold shock treatment). NaCl treatments were performed using three periods of exposure to high salt concentration: 30 min, 24 h and 29 h. On the other hand, the cold shock treatment consisted of a 48 h-cold exposure. For "switch-off" experiments, 24 h-NaCl treated mycelia were returned for 5 h to control medium (hereafter designated as NaCl→CM). All treated and untreated mycelia were harvested after 30-days' growth.

Total RNA for Northern Blot analysis was extracted with a LiCl method [52] from *T. borchii *mycelia grown for: a) 30 min, 24 h and 29 h or under of NaCl treatment; b) 48 h of cold treatment; c) 5 h on CM after 24 h of NaCl treatment and d) 30 days at 24°C on CM.

Heat-denatured total RNAs (10 *μ*g for each sample) were fractionated on 1.8% formaldehyde-agarose gels and transferred onto a Hybond membrane (Amersham) by capillary elution. A *TbDHN1 *cDNA probe was obtained by PCR amplification on M6G10 EST as previously described and hybridized to RNA blots containing size-fractionated RNA.

The random-priming labelling of 30 ng DNA probe was carried out in the presence of [^32^P]dCTP. Stringent washes were performed at 65°C with 0.5X SSC and 0.1% (w/v) SDS. Each filter was then de-hybridized and probed with *T*. *borchii *28S ribosomal DNA (rDNA) to estimate the level of total RNA loaded in each lane. Dried filters were exposed to the phosphor screen for 24 hours. Signals were quantified with a Personal Imager FX using Quantity One (Version 4.5.0) software (Biorad). Normalization for differences in loading, labelling and hybridisation were made based on the 28S ribosomal signal. Normalized signals of all treatments were compared with the control signals. Expression data are representative of three independent experiments.

Genomic DNA samples for gel blot analysis (10 μg each) were obtained from mycelia, according to Henrion et al. [53], digested with *HindIII*, *KpnI *and *SmaI *and size fractionated on a 0.8% agarose gel. A chemiluminescent labelling kit (ECL direct nucleic acids labelling and detection system, Amersham) was used for labelling *TbDHN1 *genomic probe; blotting onto Hybond-N (Amersham Pharmacia Biotech), pre-hybridization, hybridisation and stringent washing were conducted according to the manufacturer's instructions.

## List of abbreviations

DHN: dehydrin

LEA: late embryogenesis abundant

TIGR: The Institute for Genomic Research

EST: expressed sequence tag

CSP: conserved signature pattern

## Authors' contributions

SA carried out experimental analyses and drafted the manuscript. SA and SG participated in data analyses and wrote the manuscript. SA and PB participated in study design and coordination. All authors contributed to and approved the final version of the manuscript.

## Figures and Tables

**Table 1:** Fungal hits found by homology search using *TbDHN1 *as query sequence. *Blast *searches were conducted with *TbDHN1 *nucleotide and amino acid sequences against a local database including records from the NCBI non-redundant, the Swiss-Protein and the Broad Institute fungal databases. All records are intended to be NCBI Protein Database entries, with exception of SNU00161 (The Broad Institute Accession Number) and Q7S6B0 (Swiss Protein Database Accession Number).

**Accession number**	**Biological role**	***organism***	**Identities (%)**	**E-value**	**Sequence found by**
SNU00161	predicted protein	*Stagonospora nodorum*	41	6e-50	blastp
EAA70367	hypothetical protein FG10051	*Gibberella zeae*	40	4e-47	blastx
CAD70810	putative protein	*Neurospora crassa*	39	9e-44	blastx
EAA55454	hypothetical protein MG09261.4	*Magnaporthe grisea*	35	7e-39	blastx
EAA62484	hypothetical protein AN5324.2	*Aspergillus nidulans*	37	1e-37	blastx
EAL89059	conserved hypothetical protein	*Aspergillus fumigatus*	31	3e-35	blastx
EAL84332	conserved hypothetical protein	*Aspergillus fumigatus*	38	2e-27	blastx
EAK94850	DHN6*	*Candida albicans*	32	5e-21	blastx
EAK94909	DHN*	*Candida albicans*	39	8e-21	blastx
EAA54716	hypothetical protein MG05507.4	*Magnaporthe grisea*	28	1e-17	blastx
EAL87020	hypothetical protein Afu7g04520	*Aspergillus fumigatus*	28	2e-16	blastx
Q7S6B0	Predicted protein	*Neurospora crassa*	26	0.009	blastp

*Putative identifications provided by automatical annotation of *C. albicans *genome.

**Table 2:** Physiochemical analysis of TbDHN1 and its homolog sequences. Amino acid compositions, percentages of low complexity regions, percentages of unstructured polypeptide and hydropathy profiles for TbDHN1 and its homologs. All figures are intended as percentages.

	**Cys**	**Gly**	**Ser**	**Thr**	**Trp**	L.C.R.*	**Random coil**	**Hydropathy profile**
**TbDHN1 *T. borchii***	0.0	19.7	10.5	22.8	0.0	57	76.1	
**CAD70810 *N. crassa***	0.0	17.1	9.8	12.8	0.0	28	86.2	
**Q7S6B0 *N. crassa***	0.0	8.5	9.3	5.4	0.0	0	89.2	
**EAA62484 *A. nidulans***	0.0	14.6	11.8	14.6	0.0	22	88.6	
**EAA55454 *M. grisea***	0.0	16.5	12.9	14.1	0.0	44	82.3	
**EAA54716 *M. grisea***	0.4	16.3	12.6	9.1	0.2	25	87.0	
**SNU00161 *S. nodorum***	0.0	20.6	7.8	18.8	0.0	52	87.2	
**EAL89059 *A. fumigatus***	0.0	12.0	8.7	13.1	0.0	24	94.7	
**EAL84332 *A. fumigatus***	0.0	15.0	11.8	13.4	0.0	37	90.2	
**EAL87020 *A. fumigatus***	0.0	12.6	11.6	11.6	0.0	5	95.6	
**EAK94909 *C. albicans***	0.0	23.5	16.0	14.2	0.0	56	70.8	
**EAK94850 *C. albicans***	0.0	23.6	15.8	14.3	0.0	55	63.9	
**EAA70367 *G. zeae***	0.0	16.0	14.2	12.9	0.0	45	92.5	

*L.C.R., Low Complexity Regions

**Table 3:** LEA proteins used for phylogenetic analysis. Amino acid sequences corresponding to LEA proteins were retrieved from the Swiss-Protein database.

**Accession number**^**a**^	**Organism**	**Classified as**
P04568	*Triticum aestivum*	LEA 1
P09443	*Gossypium hirsutum*	LEA 1
P11573	*Raphanus sativus*	LEA 1
P17639	*Daucus carota*	LEA 1
P22701	*Triticum aestivum*	LEA 1
P46514	*Helianthus annuus*	LEA 1
P46517	*Zea mays*	LEA 1
P46520	*Oryza sativa*	LEA 1
Q02973	*Arabidopsis thaliana*	LEA 1
Q05190	*Hordeum vulgare*	LEA 1
Q05191	*Hordeum vulgare*	LEA 1
Q07187	*Arabidopsis thaliana*	LEA 1
O04232	*Solanum tuberosum*	LEA 2
O22623	*Vaccinium corymbosum*	LEA 2
O48622	*Spinacia oleracea*	LEA 2
O65216	*Triticum aestivum*	LEA 2
P12253	*Oryza sativa*	LEA 2
P12950	*Zea mays*	LEA 2
P22239	*Craterostigma plantagineum*	LEA 2
P22240	*Lycopersicon esculentum*	LEA 2
P28639	*Pisum sativum*	LEA 2
P42758	*Arabidopsis thaliana*	LEA 2
Q07322	*Daucus carota*	LEA 2
Q9ZTR5*	*Hordeum vulgare*	LEA 2
O49816	*Cicer arietinum*	LEA 3
P13934	*Brassica napus*	LEA 3
P13939	*Gossypium hirsutum*	LEA 3
P14928	*Hordeum vulgare*	LEA 3
P20075	*Daucus carota*	LEA 3
P23283	*Craterostigma plantagineum*	LEA 3
Q39058	*Arabidopsis thaliana*	LEA 3
Q39873	*Glycine max*	LEA 3
Q40696	*Oryza sativa*	LEA 3
Q40869	*Picea glauca*	LEA 3
Q40929	*Pseudotsuga menziesii*	LEA 3
Q41060	*Pisum sativum*	LEA 3
Q00002	*Alernaria alternata*^*b*^	LEA 3
Q5UUY7	*Antonospora locustae*^*b*^	LEA 3
Q6FVF6	*Candida glabrata*^*b*^	LEA 3
Q6BSI4	*Debaryomyces hansenii*^*b*^	LEA 3
Q8SVY9	*Encephalitozoon cuniculi*^*b*^	LEA 3
Q6CP34	*Kluyveromyces lactis*^*b*^	LEA 3
Q7S1U2	*Neurospora crassa*^*b*^	LEA 3
Q7SH94	*Neurospora crassa*^*b*^	LEA 3
Q9Y7X6	*Schizosaccharomyces pombe*^*b*^	LEA 3
Q6C0I4	*Yarrowia lipolytica*^*b*^	LEA 3
Q6C052	*Yarrowia lipolytica*^*b*^	LEA 3
Q6C800	*Yarrowia lipolytica*^*b*^	LEA 3

^a ^Swiss Protein Database Accession Number; ^b ^Fungal organisms; *This is DHN6 from *Hordeum vulgare *(corresponding to GenBank: AAD02257) which represents the first hit of known biological role found by *blastx *using *TbDHN1 *as query sequence.
